# Retraction Note: Up-regulated miR-199a-5p in gastric cancer functions as an oncogene and targets klotho

**DOI:** 10.1186/s12885-022-09875-9

**Published:** 2022-07-14

**Authors:** Xu-Jun He, Ying-Yu Ma, Sheng Yu, Xiao-Ting Jiang, Yi-Ding Lu, Liang Tao, Hua-Ping Wang, Zhi-Ming Hu, Hou-Quan Tao

**Affiliations:** 1 Key Laboratory of Gastroenterology of Zhejiang Province, Zhejiang Provincial People’s Hospital, Hangzhou, 310014 China; 2 Wenzhou Medical College, Wenzhou, 310025 Zhejiang Province China; 3 Clinical Laboratory, Zhejiang Provincial People’s Hospital, Hangzhou, 310014 China; 4 Department of Surgery, Haining No.3 People’s Hospital, Haining, 314408 Zhejiang Province China; 5 Department of Gastrointestinal Surgery, Zhejiang Provincial People’s Hospital, Hangzhou, 310014 China

**Retraction Note: BMC Cancer 14, 218 (2014)**

**https://doi.org/10.1186/1471-2407-14-218**

The Editors have retracted this article because of significant concerns regarding figure 2 presented in this work, which questions the integrity of the data. The Editors therefore no longer have confidence in the integrity of the data in this article. None of the authors agree to this retraction.

